# Physicochemical Properties of Egg-Box-Mediated Hydrogels with Transiently Decreased pH Employing Carbonated Water

**DOI:** 10.1021/acsomega.2c07552

**Published:** 2023-02-16

**Authors:** Ryota Teshima, Shigehito Osawa, Yayoi Kawano, Takehisa Hanawa, Akihiko Kikuchi, Hidenori Otsuka

**Affiliations:** † Department of Chemistry, Graduate School of Science, Tokyo University of Science, 1-3 Kagurazaka, Shinjuku, Tokyo 162-8601, Japan; ‡ Department of Applied Chemistry, Faculty of Science, Tokyo University of Science, 1-3 Kagurazaka, Shinjuku, Tokyo 162-8601, Japan; § Water Frontier Research Center (WaTUS), Research Institute for Science and Technology, Tokyo University of Science, 1-3 Kagurazaka, Shinjuku, Tokyo 162-8601, Japan; ∥ Department of Pharmacy, Faculty of Pharmaceutical Sciences, Tokyo University of Science, 2641 Yamazaki, Noda, Chiba 278-8510, Japan; ⊥ Department of Materials Science and Technology, Faculty of Advanced Engineering, Tokyo University of Science, 6-3-1 Niijuku, Katsushika, Tokyo 125-8585, Japan

## Abstract

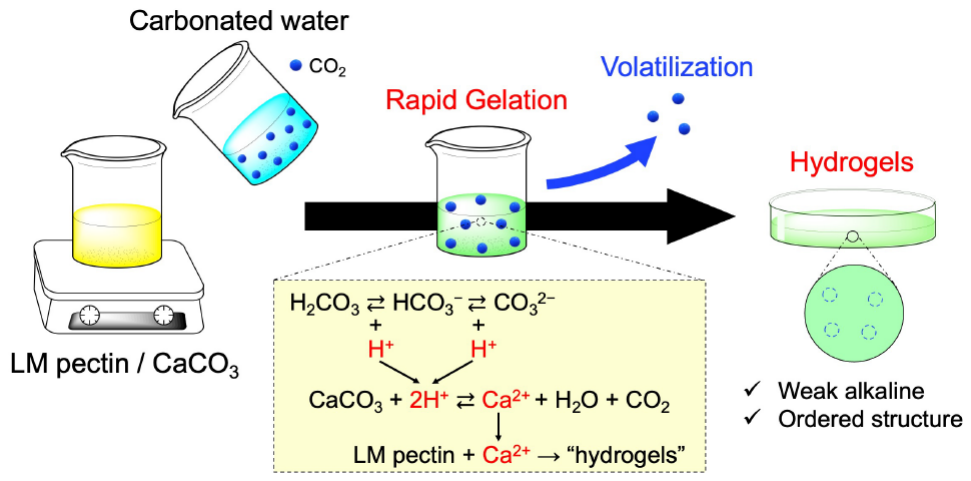

Anionic polysaccharides, including low-methoxy (LM) pectin, are extensively used in biomaterial applications owing to their safety, biocompatibility, and feasibility in constructing supramolecular assemblies by forming egg-box structures with divalent cations. Mixing an LM pectin solution with CaCO_3_ spontaneously forms a hydrogel. The gelation behavior can be controlled by adding an acidic compound to change the solubility of CaCO_3_. CO_2_ is used as the acidic agent and can be easily removed after gelation, thereby reducing the acidity of the final hydrogel. However, CO_2_ addition has been controlled under varied thermodynamical conditions; therefore, specific CO_2_ effects on gelation are not necessarily visualized. To evaluate the CO_2_ impact on the final hydrogel, which would be extended to control hydrogel properties further, we utilized carbonated water to supply CO_2_ into the gelation mixture without changing its thermodynamic conditions. The addition of the carbonated water accelerated gelation and significantly increased the mechanical strength, promoting cross-linking. However, the CO_2_ volatilized into the atmosphere, and the final hydrogel became more alkaline than that without the carbonated water, probably because a considerable amount of the carboxy group was consumed for cross-linking. Moreover, when aerogels were prepared from the hydrogels with carbonated water, they exhibited highly ordered networks of elongated porosity in scanning electron microscopy, proposing an intrinsic structural change by CO_2_ in the carbonated water. We also controlled the pH and strength of the final hydrogels by changing the CO_2_ amounts in the carbonated water added, thereby validating the significant effect of CO_2_ on hydrogel properties and the feasibility of using carbonated water.

## Introduction

Soft biomaterials based on polysaccharides have attracted attention because of their biological recognizability, biocompatibility, and unique self-assembling properties.^1−3^ Among polysaccharides, anionic polysaccharides, such as alginates and low-methoxy (LM) pectin, form stable complexes with divalent cations, such as Ca^2+^, i.e., egg-box structures, eventually forming supramolecular assemblies and leading to the gelation of hydrogels.^4−6^ Owing to their biocompatibility, egg-box-mediated hydrogels have been utilized in various life science applications, from drug delivery systems to tissue engineering.^7−9^ When alginates and LM pectin solutions are mixed with CaCl_2_ solutions, cross-linking with Ca^2+^ instantly occurs, forming hydrogel beads.^10,11^ However, when alginates and LM pectin solutions are mixed with CaCO_3_ to provide Ca^2+^ gradually, shape-controlled gelation occurs.^12,13^ The methods of providing Ca^2+^ determine the properties of the egg-box-mediated supramolecules.

In hydrogel preparation, the gelation rate, strength, and pH of the obtained hydrogel are closely related to the applicability of the gel. These parameters can be controlled in alginate/CaCO_3_ and LM pectin/CaCO_3_ hydrogels by changing the pH of the gel precursor mixture. Notably, the solubility of CaCO_3_ is superior at a lower pH, facilitating the supply of Ca^2+^ to the polymer chain. Therefore, glucono-δ-lactone (GDL) is often added to mixed solutions of alginate/CaCO_3_ and LM pectin/CaCO_3_ because of its high biocompatibility.^12−15^ The addition of GDL accelerates gelation and increases the strength of the hydrogel because protons are provided by GDL hydrolysis. The physicochemical property of the final hydrogel can easily be predicated as adding more GDL induces a more rapid sol–gel transition and yields a harder hydrogel with a lower pH. However, the low pH limits the applicability of the hydrogels. Acidic agents remaining in the hydrogels account for the low pH.

Several studies have used CO_2_ to facilitate or trigger hydrogel fabrication based on pH changes, expecting that the CO_2_ would be released from the hydrogel into the atmosphere.^16−23^ Some studies have reported that the formation of LM pectin/CaCO_3_ hydrogels^16^ and alginate/CaCO_3_ hydrogels^17−19^ can be stimulated by employing high-pressure or supercritical CO_2_ to dissolve a considerable amount of CO_2_ in polymer-containing gelation mixtures. In these methods, the temperature and pressure of CO_2_ are essential parameters to control the physicochemical properties of the final hydrogels. However, the effect of CO_2_ as an acidic agent on the properties of hydrogels has not yet been elucidated because these hydrogels are formed under varied thermodynamical conditions. In other words, there is still a question of the existence of CO_2_, which would finally go out from the hydrogel, inherently having an influence on the gelation process or properties of the finally obtained hydrogels. A precise understanding of the effect of CO_2_ would promote the controllability of the final hydrogel’s properties, including the pH and strength, which are crucial parameters for biomedical applications. Notably, applying hydrogels to control these parameters under ambient temperature and pressure would be suitable.

**Figure 1:**
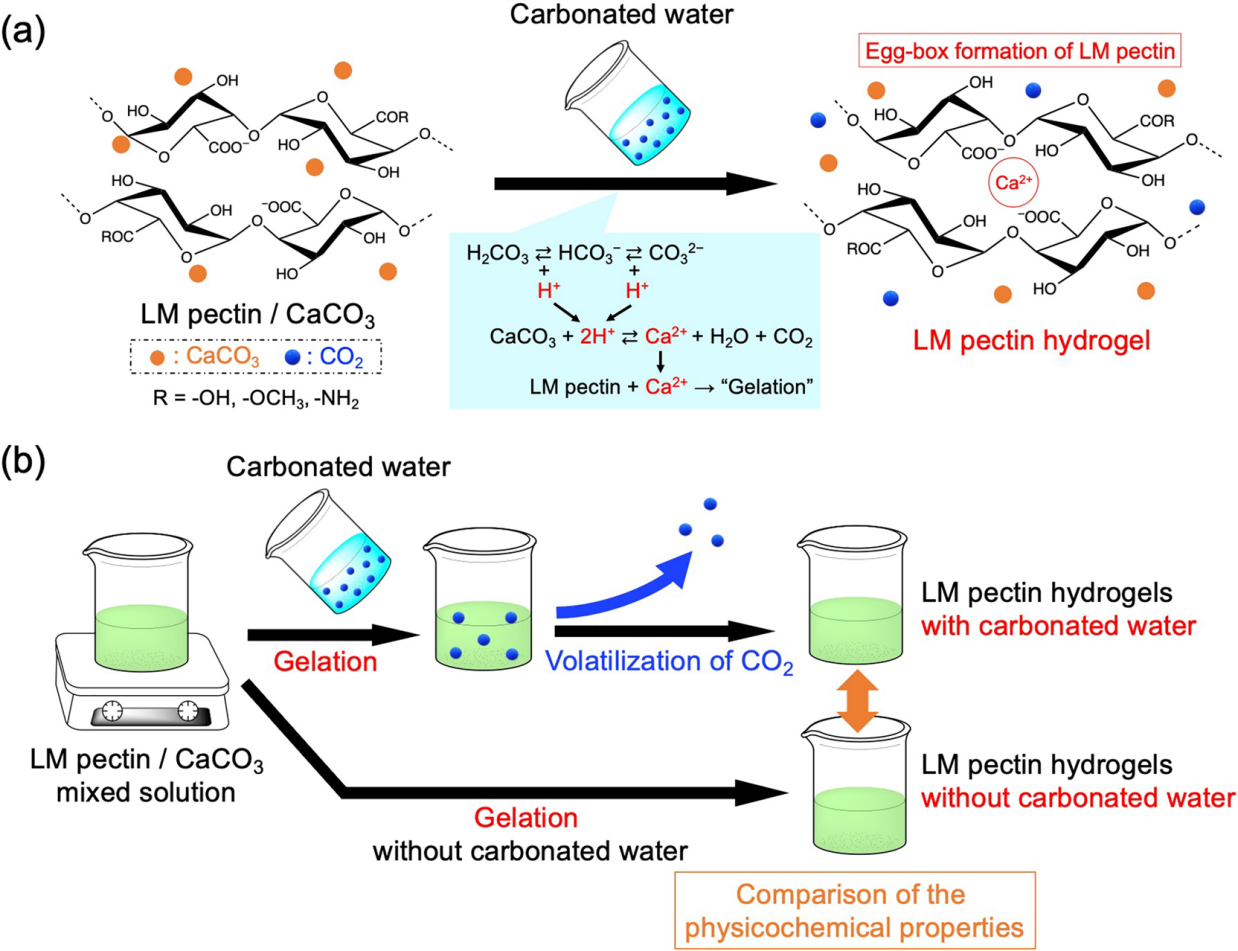
Schematic representation of this study. (a) Gelation kinetics of LM pectin/CaCO_3_ with carbonated water. (b) Comparison of physicochemical properties of the LM pectin hydrogels with and without carbonated water.

In this study, LM pectin hydrogels were prepared using carbonated water to investigate the influence of adding CO_2_ as the acidic agent (Figure 1a). We have developed a method using carbonated water to supply carbonate ions and CO_2_ to a gelation mixture; this triggered the gelation of the mixed alginate/CaCO_3_ solution under ambient pressure and temperature without requiring precise pressure and temperature controls.^20,21^ Unlike the alginate/CaCO_3_ evaluated in our previous report,^20,21^ LM pectin/CaCO_3_ undergoes sol–gel transition without adding acidic agents.^14^ Therefore, the effects of CO_2_ could be observed by comparing the LM pectin/CaCO_3_ hydrogels with and without the carbonated water (Figure 1b). Herein, we evaluated the gelation behaviors and the physicochemical properties of the resulting hydrogels on the LM pectin/CaCO_3_ mixture with or without carbonated water.

## Experimental Section

### Materials

LM pectin (GENU pectin LM-102AS-J) was provided by Sansho Co. Ltd. (Osaka, Japan). The average degree of methoxylation was ∼30% and that of amidation was ∼19%. CaCO_3_ and GDL were purchased from FUJIFILM Wako Pure Chemical Corp. (Osaka, Japan). Bromothymol blue (BTB) solution (0.04%) was purchased from Kanto Chemical Co. Inc. (Tokyo, Japan). SodaStream Spirit One Touch (SodaStream International Ltd., Israel) was employed to produce the carbonated water. The water used in this study was purified using a Milli-Q system (Nihon Millipore Co., Tokyo, Japan). All reagents were used without further purification. Dulbecco’s modified Eagle medium (DMEM), penicillin–streptomycin (PS), and fetal bovine serum (FBS) were purchased from Life Technologies Corp. (Grand Island, NY, USA). Cell Counting Kit-8, Calcein-AM, and Ethidium homodimer (EthD-1) were purchased from DOJINDO Lab. Co. (Kumamoto, Japan).

### Preparation of Carbonated Water

Carbonated water was prepared based on the protocol of SodaStream Spirit One Touch. First, a bottle (500 mL) was filled with 450 mL of purified water and cooled in a refrigerator to approximately 6 °C. After that, the bottle was attached to the SodaStream, and CO_2_ gas was sealed into the purified water by setting the CO_2_ intensity to “medium.” The obtained carbonated water was used immediately in the experiment without storing it.

### Preparation of LM Pectin Hydrogels with and without Carbonated Water

LM pectin (0.40 g) was completely dissolved in 20 mL of purified water using a magnetic stirrer. Then, CaCO_3_ (0.05 g) was suspended in 5 mL of purified water and added to the pectin solution under continuous stirring. Immediately after adding the CaCO_3_ suspension, carbonated water (10 mL) was added to the solution and stirred at 600 rpm for 10 s to prepare hydrogels. The LM pectin hydrogels without the carbonated water were prepared similarly, except by adding 10 mL of purified water instead of the carbonated water. The hydrogels prepared with the carbonated water are abbreviated as Pec-Ca-CW, and those prepared without the carbonated water are abbreviated as Pec-Ca.

### Gelation Time Measurements of LM Pectin Hydrogels

The gelation time was determined by dispensing 2 mL of the gel precursor solution into sample bottles with inner diameters of 18 mm, and the bottles were inverted. The starting time (*t* = 0) began when the carbonated water or purified water was added to the solution.

### Stress–Strain Measurements of LM Pectin Hydrogels

The stress–strain characteristics of the LM pectin hydrogels were measured using a creep meter (RE2-33005C(XZ), Yamaden Co., Ltd., Tokyo, Japan). First, 20 mL of the gel precursor solution was dispensed into stainless steel Petri dishes (50 mm diameter) and incubated for 24 h at approximately 23 °C, yielding 9–10 mm hydrogels. Next, the hydrogel breaking strength was measured via compression tests using a plunger of 20 mm diameter.

The stress–strain characteristics of the Pec-Ca-CW with different strain directions were measured as follows: cubic molds (18 × 18 × 18 mm) were prepared using glass slides, and 6 mL of the gel precursor solution was dispensed into the molds. The hydrogels were incubated for 2 days at approximately 23 °C, and the breaking strength with different strain directions was measured via compression tests using a plunger of 12.7 mm diameter. The breaking energy was calculated from the area of the stress–strain curve up to 60% strain.

### Water Content Measurements

The LM pectin hydrogels were prepared using the mentioned method and dispensed into plastic Petri dishes (90 mm diameter, gel thickness = 3.26 ± 0.35 mm). Hydrogel disks were removed from the plastic Petri dishes using a 16 mm-diameter cork borer, and the hydrogel disks were lyophilized. The water content was calculated based on the initial weight (*W*_0_) and the post-lyophilization weight (*W*_d_) of the hydrogel disks using eq 1
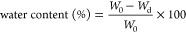
1

### Visualization of pH Change of LM Pectin Hydrogels by BTB

The LM pectin hydrogels were stained with BTB: LM pectin (0.40 g) was completely dissolved in 20 mL of purified water using a magnetic stirrer. Separately, 0.05 g of CaCO_3_ was suspended in 5 mL of purified water. First, these solutions were mixed, and then 1 mL of BTB solution was added to the solution under continuous stirring. Immediately, 10 mL of carbonated water was added to the solution and stirred at 600 rpm for 10 s. Then, 5 mL of the gel precursor solution was transferred into sample bottles with inner diameters of 25 mm, and a color change was observed for 2 days.

### Surface pH Measurements of LM Pectin Hydrogels

The surface pH values of the hydrogels were measured using a previously reported method.^20^ A pH meter with a flat electrode (HORIBA, LAQUAtwin pH-33B, Kyoto, Japan) was employed to measure the surface pH. The LM pectin hydrogels were prepared using the above-mentioned method and dispensed into plastic Petri dishes (90 mm diameter, gel thickness = 3.26 ± 0.35 mm). The surface of the hydrogel in contact with the atmosphere was defined as the front surface, whereas that in contact with the bottom of the Petri dish was defined as the back surface. After 30, 90, 150, 360, and 480 min incubation, the hydrogel disks were removed from the plastic Petri dishes using a 16 mm-diameter cork borer, and the pH values of the front and back surfaces were measured. The starting time (*t* = 0) was when the carbonated water was added. Then, a flat electrode (HORIBA, 0040N-10D, Kyoto, Japan) was used to measure the surface pH of the hydrogel in the bottle tube.

### SEM Observations of LM Pectin Hydrogels

The prepared hydrogels were imaged using scanning electron microscopy (SEM). The gel precursor solution (30 mL) was dispensed into plastic Petri dishes (90 mm diameter) and left for 24 h at approximately 23 °C. The hydrogel disks were then removed using a 16 mm-diameter cork borer, and the disks were lyophilized. After that, hydrogel cross sections were prepared using a razor blade. Finally, the cross sections of the hydrogels were sputter-coated with platinum or gold and imaged using SEM (JSM-6490LA or JSM-6060LA, JEOL, Ltd., Tokyo, Japan) at an accelerated voltage of 5 and 10 kV.

### Biocompatibility of LM Pectin Hydrogels

Normal human dermal fibroblasts (NHDFs) were purchased from Cosmo Bio Co., Ltd (Tokyo, Japan). The LM pectin hydrogels were prepared using the method mentioned above, and the hydrogel disks were prepared (11.5 mm diameter, gel thickness = 3.26 ± 0.35 mm). The hydrogel disks were immersed in 45 mL of DMEM (containing 2% PS) for 2 days to exchange the solvent. NHDFs (passage 11) were seeded in 24-well plates (1.42 × 10^4^ cells/well, for CCK-8 assay) and 12-well plates (2.84 × 10^4^ cells/well, for Live/Dead assay) and cultured in 1 mL of DMEM (containing 10% FBS and 2% PS) for 2 days at 37 °C and 5% CO_2_. Then, a gel disk (1 disk/well) was added to the culture with the DMEM exchange. After 1 day, cell viability was evaluated by CCK-8 assay and Live/Dead assay. For the CCK-8 assay, after all media were collected, a 10-fold dilution of CCK solution was added and incubated for 60 min at 37 °C. Then, the absorbance at 450 nm was measured. For the Live/Dead assay, 4 μL of Calcein-AM and 2 μL of EthD-1 were added and incubated for 30 min at 37 °C. Then, the cells were observed under an Axio Observer D1 microscope (Carl Zeiss Co, Ltd., Oberkochen, Germany).

### Statistical Analysis

Student’s *t*-test was used in the cell study. A value of *p* < 0.05 was considered statistically significant.

## Results and Discussion

### Preparation of LM Pectin Hydrogels with and without Carbonated Water

**Figure 2:**
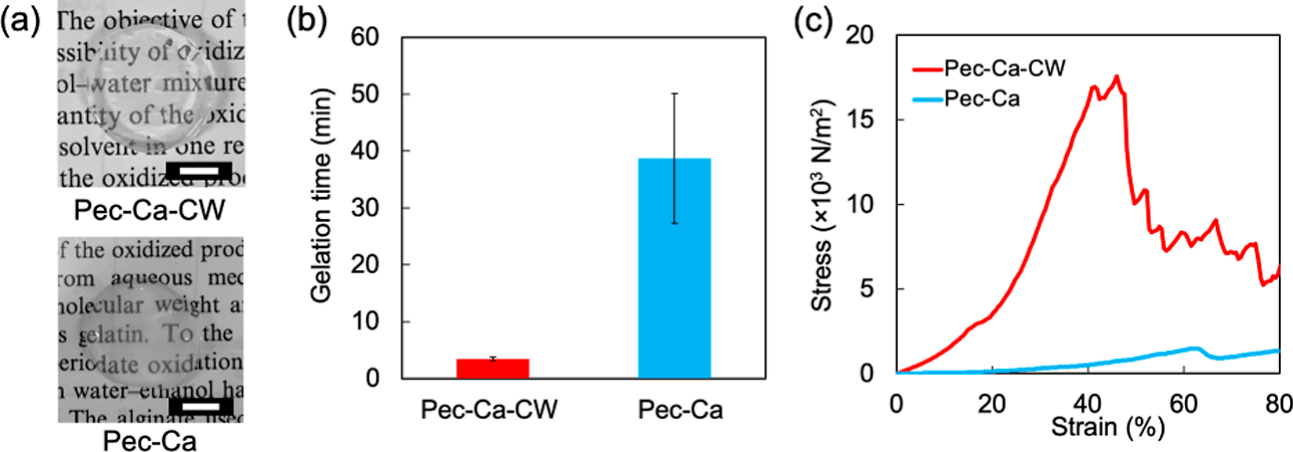
Preparation and characterization of LM pectin hydrogels prepared with (Pec-Ca-CW) and without (Pec-Ca) carbonated water. (a) Overhead photographs of Pec-Ca-CW and Pec-Ca. The scale bar is 5 mm. (b) Gelation times of Pec-Ca-CW and Pec-Ca. The values are expressed as the mean ± SD obtained from four experiments. (c) Stress–strain curves of Pec-Ca-CW and Pec-Ca.

Both LM pectin/CaCO_3_ solutions with and without the carbonated water successfully formed transparent hydrogels, termed Pec-Ca-CW and Pec-Ca, respectively (Figure 2a). Note that Pec-Ca-CW seems to have a little higher transparency than Pec-Ca. Dissolution behaviors of the dispersed CaCO_3_ were different during gelation periods between Pec-Ca-CW and Pec-Ca. The gelation periods were evaluated by tube inversion tests and significantly shortened by adding carbonated water (Figure 2b). Unlike Pec-Ca, Pec-Ca-CW could be picked up with tweezers (Figure S1), suggesting that adding the carbonated water significantly changed the hydrogels’ strength. The stress–strain curve measurement showed that Pec-Ca-CW had a higher strength than Pec-Ca (Figure 2c). The lower breaking strain of Pec-Ca-CW further suggests that the network structure of LM pectin/CaCO_3_ was wholly changed by adding the carbonated water. The pH of the carbonated water used in these evaluations was 3.64 ± 0.06 (*n* = 3, mean ± SD), containing CO_2_ over saturation. The results mentioned above indicated that the addition of the carbonated water promoted the hydrogelation of the LM pectin/CaCO_3_ solutions based on the dissolution of CaCO_3_, similar to the addition of GDL as the acidic agent reported in the literature.^12−15^ The water contents of the final hydrogels were the same regardless of the addition of the carbonated water (Table 1), suggesting that the final components of the hydrogels did not change by adding the carbonated water because CO_2_ was spontaneously released into the atmosphere.

**Table 1:** Water Contents of LM Pectin Hydrogels

hydrogels	water content (%)^a^
Pec-Ca-CW	98.3 ± 0.14
Pec-Ca	98.5 ± 0.05

a Values are expressed as the mean ± SD obtained from the three samples.

### Surface pH Changes of LM Pectin Hydrogels Prepared with Carbonated Water

**Figure 3:**
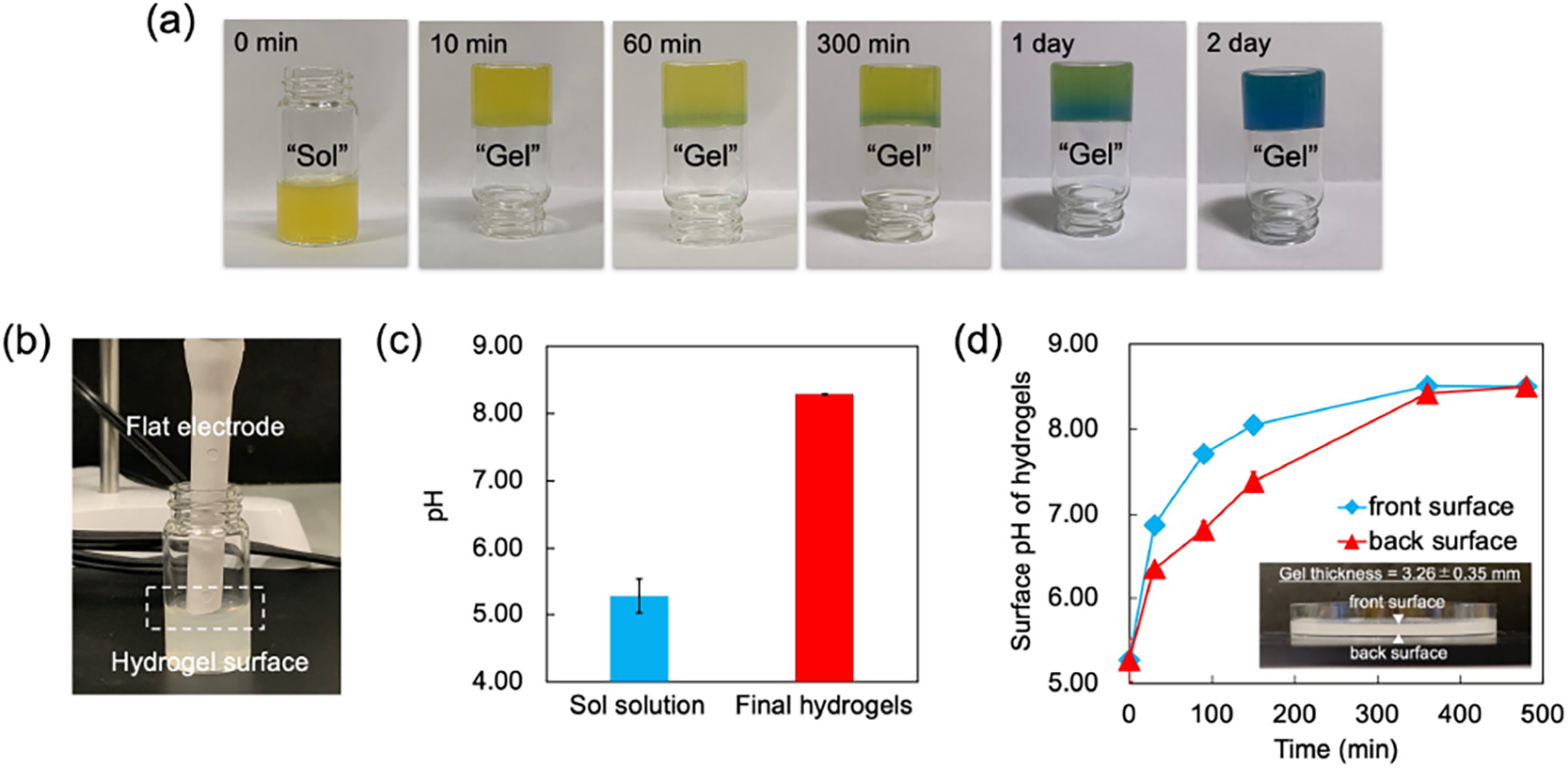
Surface pH of LM pectin hydrogels. (a) pH changes in Pec-Ca-CW containing BTB over time (*t* = 0 refers to the time when carbonated water is added). (b) Measurement of surface pH of Pec-Ca-CW in a vial tube. (c) Initial pH and final pH of Pec-Ca-CW expressed as the mean ± SD obtained from three experiments. (d) Surface pH changes in Pec-Ca-CW of 3.26 ± 0.35 mm thickness expressed as the mean ± SD obtained from three or four experiments.

The pH of Pec-Ca-CW was monitored to elucidate the gelation behavior and kinetics of the CO_2_ released from Pec-Ca-CW. We hypothesized that the volatilization of CO_2_ from the hydrogels would continuously reduce their acidity, thereby changing the pH. For a precise and visible evaluation of the pH changes in the hydrogels, we added BTB to the gelation mixture as a pH indicator that appears yellow, green, and blue in acidic, neutral, and alkaline conditions, respectively. The hydrogel was acidic immediately after adding the carbonated water, and the solution transitioned to a gel within a short time, as shown in Figure 3a. The color of the obtained hydrogel surface turned slightly blue after 60 min, and the yellow color gradually retreated from the gel surface over time. The closer to the surface, the bluer the color was, reflecting the pH gradient in the hydrogel, and the pH of the hydrogel surface at 2 days, measured with a flat electrode (Figure 3b), was 8.2 (Figure 3c). This color change indicates that the concentrations of CO_2_ and carbonate ions decreased from the top surface via gaseous exchange with the atmosphere. At 2 days, the hydrogel became entirely blue, suggesting that CO_2_ was eliminated from the hydrogel in the bottle.

Then, the pH of Pec-Ca-CW was measured for 480 min to quantitatively evaluate its pH change behavior. The difference in pH was compared between the hydrogel surface exposed to the atmosphere, termed as the front surface, and that to the dish bottom, termed as the back surface, under the assumption that the pH value changes from the front surface. The surface pH changes were evaluated on hydrogels with 3.26 ± 0.35 mm thickness (Figure 3d). The pH values of the front surfaces increased faster than those of the back surfaces. The diffusion of CO_2_ in the hydrogel may delay the pH change in the back surfaces, as shown in Figure 3a. Notably, no pH gradient appeared in a mixture of CaCO_3_ and carbonated water without LM pectin, implying that the carbonate ions and CO_2_ exhibited different behaviors in the hydrogels and in the presence of LM pectin. Their diffusion was assumed to be limited in the gelation mixtures and hydrogels. Although several factors, including the reaction equilibriums of carboxy groups (COOH), CaCO_3_, and Ca^2+^ and the formation of calcium carboxylates (2COO^–^–Ca^2+^), may affect the distribution, the carbonated water significantly affected the gelation kinetics and properties of the hydrogels.

### Structural Investigation of LM Pectin Hydrogels with Carbonated Water

**Figure 4:**
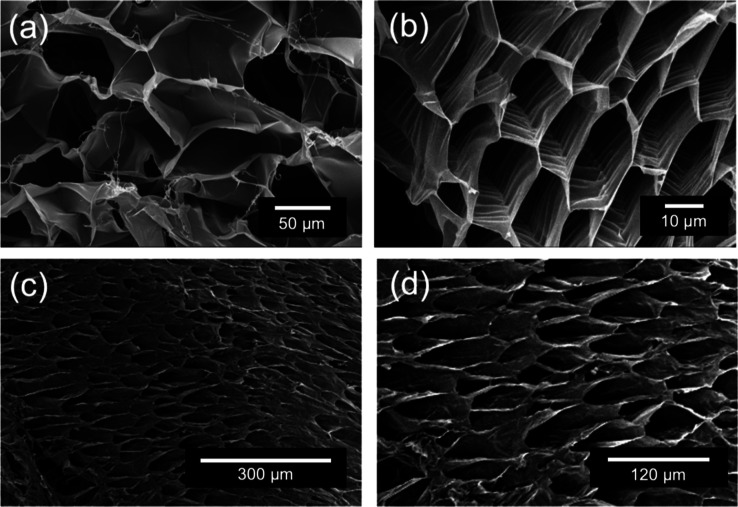
SEM images of aerogels prepared through the lyophilization of the LM pectin hydrogels. (a) Cross section of Pec-Ca (×400), (b) cross section of Pec-Ca-CW (×1400), (c) vertical cross sections of Pec-Ca-CW (×100), and (d) vertical cross sections of Pec-Ca-CW (×250).

The carbonated water significantly changed the gelation kinetics, and the resulting hydrogel, as mentioned previously, motivated us to compare the microstructure of Pec-Ca-CW with that of Pec-Ca. We prepared aerogels from Pec-Ca-CW and Pec-Ca, and their horizontal cross sections were observed using SEM (Figure 4). The aerogel prepared from Pec-Ca had a randomly cross-linked structure (Figure 4a). In contrast, the aerogel prepared from Pec-Ca-CW had a uniform and robust hexagonal structure (Figure 4b). Notably, lyophilization to prepare the aerogel was performed after incubating the LM pectin/CaCO_3_ solution for 24 h until the pH of the hydrogel converged, and CO_2_ might not have remained in the hydrogel. Significantly, the hydrogel components are the same in both cases. Therefore, the possibility of differentiating between them during the aerogel preparation process is little, and the original network structure would be inherently different in Pec-Ca-CW and Pec-Ca in the hydrogel state. Considering the significant acceleration of hydrogel formation by adding the carbonated water (Figure 2b), this result is unique because a more rapid transition generally forms a more disordered structure. This highly ordered structure may explain a bit of increased transparency of the hydrogel, as shown in Figure 2a, and the significant increase in the mechanical strength of the hydrogels, as shown in Figure 2c. The aerogel’s vertical cross sections were observed to elucidate the Pec-Ca-CW structure further. The elongated porosity was successfully observed (Figure 4c,d), suggesting that the volatilization or migration of CO_2_ leads to the physical building of the specific network structure during the hydrogel preparation process. The routes of CO_2_ migration in the hydrogels might be formed during the gelation process.

**Figure 5:**
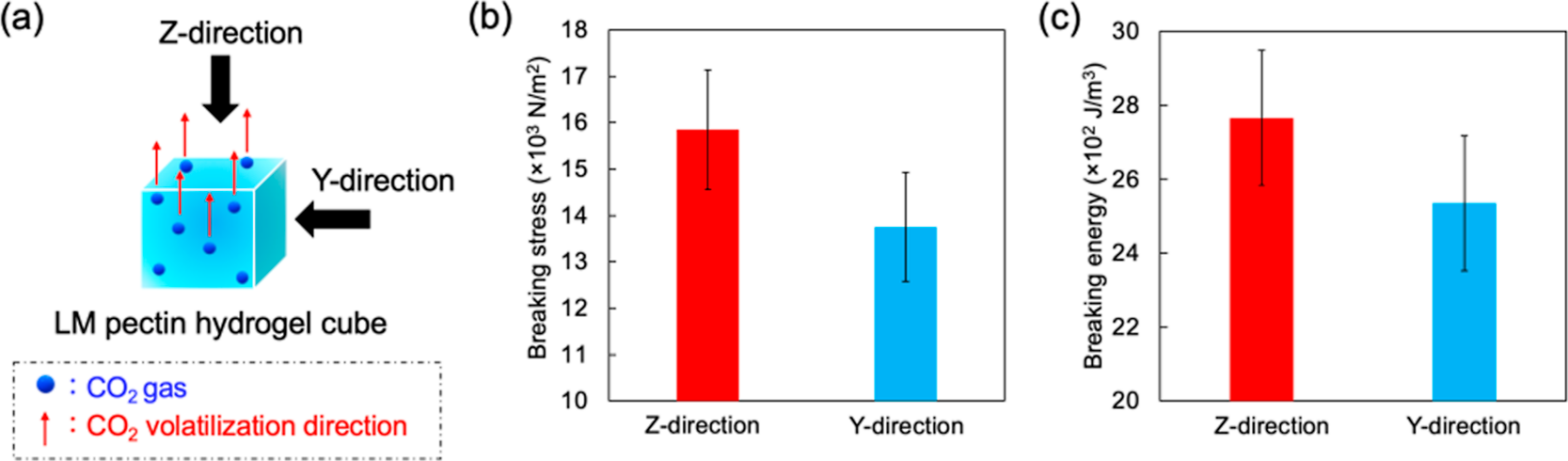
Physical anisotropy of Pec-Ca-CW. (a) Strain direction and CO_2_ volatilization direction in hydrogel cubes. (b) Breaking stress of Pec-Ca-CW in different strain directions, expressed as the mean ± SD obtained from four experiments. (c) Breaking energy of Pec-Ca-CW in different strain directions, expressed as the mean ± SD obtained from four experiments.

To further analyze the above-described specific structure of the hydrogel, we performed compression tests on a cube of Pec-Ca-CW (∼18 × 18 × 18 mm) with changing strain directions. First, the gel precursor solutions were poured into a cube mold, which could only release CO_2_ from the top surface. After releasing CO_2_, the breaking stresses for strain toward the *z*- and *y*-axis directions were measured (Figure 5a). The breaking strength toward the *z*-axis, the direction of CO_2_ volatilization, was slightly higher than that toward the *y*-axis (Figure 5b). Furthermore, more significant energy was required to break the Pec-Ca-CW cube from the *z*-axis direction than from the *y*-axis (Figure 5c, stress–strain curves are shown in Figure S2). These results imply that Pec-Ca-CW may have an anisotropic structure. This specific structure may be formed during the hydrogel preparation process and would be beneficial for various applications.^24,25^ Further, the control of the structure may extend future applications for hydrogels and aerogels.

### Final pH of LM Pectin Hydrogels and the Cross-Linking Effect of Carbonated Water

The pH of hydrogels is significant in determining the application of the synthesized hydrogels.^14,26,27^ For example, pectin hydrogels are often used as scaffolds for tissue engineering^28^ and wound dressings.^29,30^ For tissue engineering applications, the pH of the hydrogel must be adjusted to physiological levels to avoid disturbing cell proliferation and differentiation. For wound dressing applications, the pH of the hydrogel should be related to the wound-healing process.^31^

**Figure 6:**
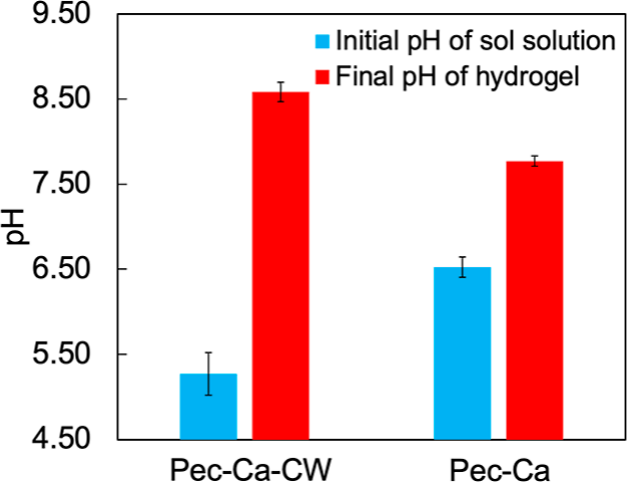
Initial and final pH values of LM pectin hydrogels. The pH of sol solution within *t* = 3 min. The pH of the final hydrogels incubated for 24 h at approximately 23 °C. Values are expressed as the mean ± SD obtained from *n* = 3–5.

During the Pec-Ca-CW preparation process, the pH transiently decreases and then gradually increases, owing to the release of CO_2_ into the atmosphere, eventually producing a small amount of alkaline hydrogel. Interestingly, Pec-Ca-CW is more alkaline than Pec-Ca, although the initial pH of the former is lower (Figure 6). This difference was not observed in the previous study on alginate/CaCO_3_ hydrogels^20^ because alginate/CaCO_3_ could not be transformed into a hydrogel without carbonated water. Pec-Ca-CW can have a higher cross-linking structure, as shown in Figure 2c. Therefore, the number of carboxylate residues not forming the egg-box structure would decrease in the prepared hydrogel with the carbonated water, resulting in more alkaline hydrogels.

**Figure 7:**
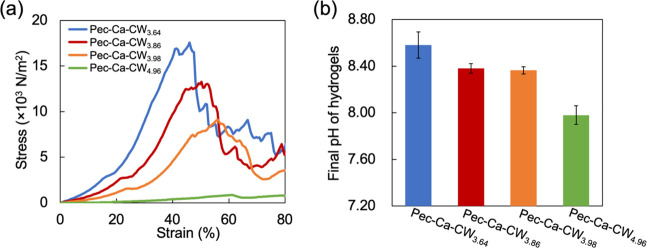
Relationship between mechanical strength and pH of Pec-Ca-CW. (a) Stress–strain curves of Pec-Ca-CW prepared with carbonated water of different pH values. (b) Final pH of Pec-Ca-CW prepared with carbonated water of different pH values. The values are expressed as the mean ± SD obtained from five experiments.

Based on the above insight, we devised a strategy to control the final pH and strength of the LM pectin/CaCO_3_ hydrogels by changing the amount of CO_2_ in the gelation mixture, i.e., the initial pH of the added carbonated water. The LM pectin/CaCO_3_ hydrogels were prepared using carbonated waters of different pH values, pH = 3.64, 3.86, 3.98, and 4.96. The resulting gels were termed Pec-Ca-CW_3.64_, Pec-Ca-CW_3.86_, Pec-Ca-CW_3.98_, and Pec-Ca-CW_4.96_, respectively. The pH of each carbonated water was prepared according to the method described in the Supporting Information. The hydrogels exhibited higher strength and lower breaking strain when carbonated water with a lower pH, i.e., the gelation mixture with the higher CO_2_ concentration, was used (Figure 7a). These results indicate that the mechanical properties of the hydrogels can be tuned by adjusting the CO_2_ concentration in the carbonated water. Furthermore, hydrogels with higher pH values were produced using carbonated waters with lower pH values (Figure 7b). These results support our speculation that carbonated water can control the physicochemical properties of the obtained gel, probably because of the difference in the number of carboxylate residues that did not form the egg-box structure.

Several attempts to adjust the pH of hydrogels have been made,^14,26,27^ and the methods of increasing the pH of LM pectin hydrogels have also been reported.^14^ Moreira et al. mixed NaOH or NaHCO_3_ into LM pectin/CaCO_3_ solutions to increase the hydrogel pH.^14^ In this context, adding higher pH substances yielded more alkaline LM pectin hydrogels. In other words, their study revealed that a gel precursor with a higher pH forms hydrogels with higher pH values. However, adding these high-pH substances decreases the gelation rate and degree of cross-linking. In this study, we succeeded in increasing the pH values of the hydrogels while achieving a contrary behavior, as shown in Figure 7. This gelation method using carbonated water may extend the future application of egg-box-mediated hydrogels.

### Biocompatibility of LM Pectin Hydrogels

**Figure 8:**
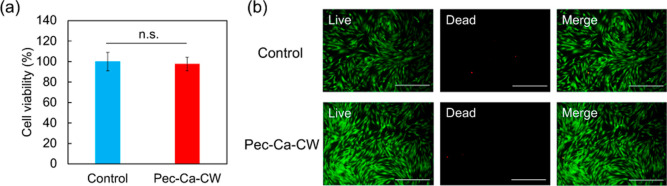
Biocompatibility of Pec-Ca-CW. (a) Cell viability after 24 h incubation with Pec-Ca-CW expressed as the mean ± SD obtained from four experiments. “n.s.” indicates not significant (*p* > 0.05). (b) Live/dead assay of NHDFs after 24 h incubation with Pec-Ca-CW. Scale bar = 500 μm.

The cytotoxicity of Pec-Ca-CW was evaluated further to extend the scope of the hydrogel as a biocompatible material. LM pectin has been highlighted to design biomaterials touching the skin, including wound dressing, owing to its inherent safety.^29,30^ Herein, we observed the cytotoxicity of Pec-Ca-CW against NHDF cells to verify that the hydrogel formation still preserves the biocompatibility of LM pectin. Pec-Ca-CW was settled on monolayer-cultured NHDF cells. Adding Pec-Ca-CW did not reduce the viability of NHDFs (Figure 8a). Furthermore, the live/dead fluorescent images (Figure 8b) support the results of the cell viability test. These results suggest that the LM pectin hydrogel prepared in this study is biocompatible and has potential application for biomaterials.

## Conclusions

The effects of CO_2_ on the physicochemical properties of LM pectin hydrogels were thoroughly elucidated using carbonated water. The gelation times of the hydrogels were significantly decreased by adding the carbonated water because this water increased the solubility of CaCO_3_ to promote cross-linking. After gelation, CO_2_ was volatilized from the hydrogel surface in contact with the atmosphere, resulting in hydrogel with a higher pH than that prepared without the carbonated water. The pH increase was due to decreased carboxyl groups in the LM pectin chain caused by cross-linking, as shown by the promoted mechanical strength. The final mechanical strength and pH of the hydrogels could be controlled by adjusting the amount of the added CO_2_. Moreover, by using carbonated water, a highly ordered network structure was formed. These results reveal that CO_2_ can change the properties and structure of egg-box-mediated hydrogels, demonstrating the advantage of using carbonated water for designing hydrogels.

## Supporting Information Available

Overhead photographs of the hydrogels handled with tweezers, stress–strain curves of Pec-Ca-CW with different strains, and pH variation of carbonated water (PDF)
