# Supplementary material for: Physicochemical Properties of Egg-Box-Mediated Hydrogels with Transiently Decreased pH Employing Carbonated Water
Source: ACS Omega. 2023 Feb 16;8(8):7800–7. doi: 10.1021/acsomega.2c07552 (PMC9979317; doi:10.1021/acsomega.2c07552)
Supplement: Supplementary file 1 — ao2c07552_si_001.pdf [file ao2c07552_si_001.pdf]

Supplementary Information

Physicochemical properties of egg-box-mediated hydrogels with transiently decreased pH employing carbonated water

Ryota Teshima,[*a] Shigehito Osawa, [bc] Yayoi Kawano, [d] Takehisa Hanawa, [d]

Akihiko Kikuchi [e] and Hidenori Otsuka [abc]

a. Department of Chemistry, Graduate School of Science, Tokyo University of Science, 1-3 Kagurazaka, Shinjuku, Tokyo 162-8601, Japan.

b. Department of Applied Chemistry, Faculty of Science, Tokyo University of Science, 1-3 Kagurazaka, Shinjuku, Tokyo 162-8601, Japan.

c. Water Frontier Science and Technology Research Center, Research Institute for Science and Technology, Tokyo University of Science, 1-3 Kagurazaka, Shinjuku, Tokyo 162-8601, Japan.

d. Department of Pharmacy, Faculty of Pharmaceutical Sciences, Tokyo University of Science, 2641 Yamazaki, Noda, Chiba 278-8510, Japan.

e. Department of Materials Science and Technology, Faculty of Advanced Engineering, Tokyo University of Science, 6-3-1 Niijuku, Katsushika, Tokyo 125-8585, Japan.

*Corresponding Author: Ryota Teshima

Department of Chemistry, Graduate School of Science, Tokyo University of Science, 1-3 Kagurazaka, Shinjuku, Tokyo 162-8601, Japan.

Email: 1322595@ed.tus.ac.jp

Supplementary Figures

Overhead photographs of the hydrogels handled with tweezers

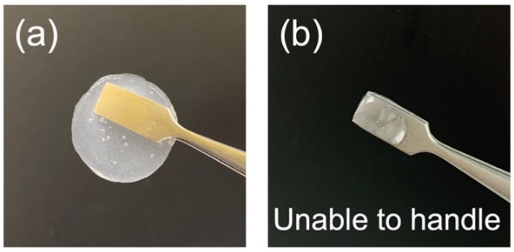

Figure S1. Photographs of the hydrogels handled with tweezers (6 mm tip width) at t = 180 min. (a) Pec-Ca-CW and (b) Pec-Ca.

Stress-strain measurements of LM pectin hydrogels with different strain

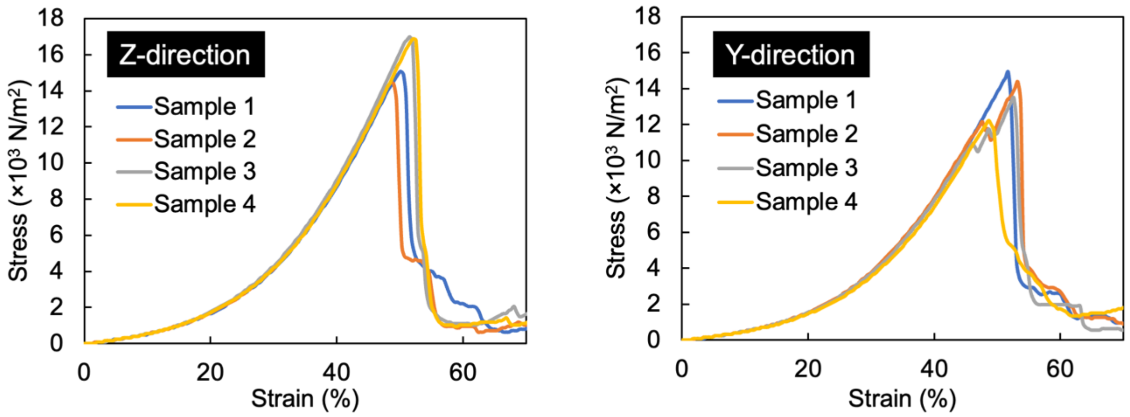

Figure S2. Stress–strain measurements of Pec-Ca-CW with different strain.

Preparation of carbonated water with different pH values

The pH of the carbonated water was controlled by stirring as follows: approximately 100 mL of the carbonated water was stirred at 600 rpm in 100 mL beaker on a cool plate (SCP-85, AS ONE, Osaka, Japan) setting to 6°C for 10–60 min. The relationship between stirring time and pH of the carbonated water is shown in Figure S3.

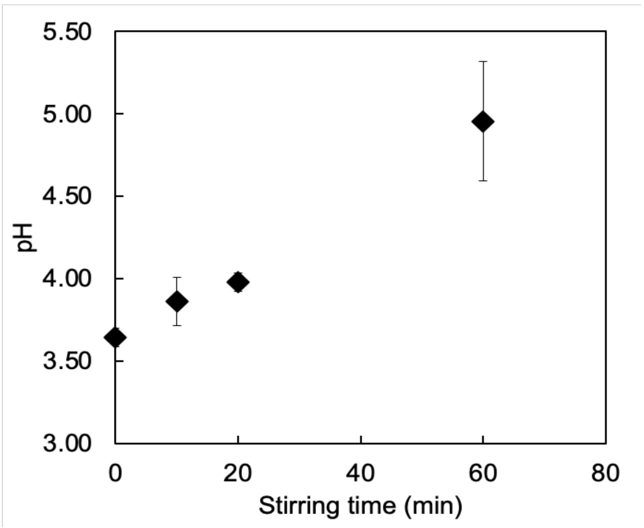

Figure S3. Relationship between pH of carbonated water and stirring time. Values are expressed as the mean ± SD obtained with n = 2-3.
